# Is It Possible to Directly Determine the Radius of a Spherical Indenter Using Force Indentation Data on Soft Samples?

**DOI:** 10.1155/2022/6463063

**Published:** 2022-02-17

**Authors:** S. V. Kontomaris, A. Stylianou, A. Malamou

**Affiliations:** ^1^Metropolitan College, Faculty of Engineering and Architecture, Athens, Greece; ^2^BioNanoTec LTD, Nicosia, Cyprus; ^3^School of Science, European University Cyprus, Cyprus; ^4^Radar Systems and Remote Sensing Lab of School of Electrical & Computer Engineering of National Technical University of Athens, Greece

## Abstract

An important factor affecting the accuracy of Young's modulus calculation in Atomic Force Microscopy (AFM) indentation experiments is the determination of the dimensions of the indenter. This procedure is usually performed using AFM calibration gratings or Scanning Electron Microscopy (SEM) imaging. However, the aforementioned procedure is frequently omitted because it requires additional equipment. In this paper, a new approach is presented that focused on the calibration of spherical indenters without the need of special equipment but instead using force indentation data on soft samples. Firstly, the question whether it is mathematically possible to simultaneously calculate the indenter's radius and the Young's modulus of the tested sample (under the restriction that the sample presents a linear elastic response) using the same force indentation data is discussed. Using a simple mathematical approach, it was proved that the aforementioned procedure is theoretically valid. In addition, to test this method in real indentation experiments agarose gels were used. Multiple measurements on different agarose gels showed that the calibration of a spherical indenter is possible and can be accurately performed. Thus, the indenter's radius and the soft sample's Young's modulus can be determined using the same force indentation data. It is also important to note that the provided accuracy is similar to the accuracy obtained when using AFM calibration gratings. The major advantage of this paper is that it provides a method for the simultaneous determination of the indenter's radius and the sample's Young's modulus without requiring any additional equipment.

## 1. Introduction

Spherical indenters are frequently used in Atomic Force Microscopy (AFM) indentation experiments on soft biological samples [1–6]. They are preferable since they usually do not cause permanent damage to the abovementioned samples during indentation (at least in cases that the indentation depth is not significantly bigger compared to the indenter's radius). The data processing when using spherical indenters is usually performed by fitting the force indentation data to the classic Hertz equation [7–9]:

(1) $$\begin{array}{c} F=\frac{4ER^{1/2}}{3\left(1-v^{2}\right)}h^{3/2}. \end{array}$$

In equation (1), *E* and *v* are the material's Young's modulus and Poisson's ratio, respectively, and *R* is the sphere's radius. Thus, the Young's modulus of the sample of interest can be accurately determined under the condition that the sample's Poisson's ratio and the indenter's radius are known. Thus, it is significant to determine the indenter's radius in order to accurately calculate the sample's Young's modulus.

In most of the cases, the radius of the indenter is measured using an AFM calibration grating [10] or using Scanning Electron Microscopy (SEM) imaging [11]. Subsequently, when *R* has been determined, the Young's modulus of the tested sample can be calculated as a fitting parameter using equation (1). This is the common approach in the literature [5, 7]. Alternatively, it is possible to obtain a force indentation curve on a sample with a well-known Young's modulus and then calculate *R* using equation (1). However, an interesting question arises: *is it mathematically possible to determine the radius of a spherical indenter using force indentation data without knowing the sample's Young's modulus?* And if yes, *is it possible to apply this method in real indentation experiments for the spherical indenter's calibration?* It is obvious that if equation (1) is used, then it is impossible to simultaneously determine *E* and *R* using only equation (1). However, it should be noted that equation (1) is valid only for small indentation depths compared to the tip radius (*h* ≪ *R*) [12]. A typical limit in the literature is *h* < *R*/10 [13]. In other words, equation (1) is just an approximation for spherical indentations since it accurately describes indentation using a paraboloid of revolution. On the contrary, regarding spherical indentations, Sneddon's equation is valid for every value of indentation depth [14]:

(2) $$\begin{array}{c} F=\frac{E}{2\left(1-v^{2}\right)}\left[\left(r_{c}^{2}+R^{2}\right)\ln\left(\frac{R+r_{c}}{R-r_{c}}\right)-2r_{c}R\right]. \end{array}$$

In equation (2), *r*_*c*_ is the radius at contact depth (*h*_*c*_) (i.e., the depth at which contact is made between the sample and the sphere). In addition,

(3) $$\begin{array}{c} \ln\left(\frac{R+r_{c}}{R-r_{c}}\right)=\frac{2h}{r_{c}}. \end{array}$$

Nevertheless, equation (2) does not directly relate the applied force on the sample to the indentation depth since the contact radius depends on the indentation depth. Thus, recently, a new equation was derived [15]:

(4) $$\begin{array}{c} F=\frac{4ER^{1/2}}{3\left(1-v^{2}\right)}h^{3/2}Z. \end{array}$$

In equation (4),

(5) $$\begin{array}{c} Z=c_{1}+\frac{3}{4}c_{2}R^{-1/2}h^{1/2}+\frac{3}{6}c_{3}R^{-3/2}h^{3/2}+\frac{3}{8}c_{4}R^{-5/2}h^{5/2}+\cdots +\frac{3}{2N}c_{N}R^{\left(3/2-N\right)}h^{N-3/2}, \end{array}$$

or

(6) $$\begin{array}{c} Z=c_{1}+\overset{N}{\underset{M=2}{\sum }}\frac{3}{2Μ}c_{M}R^{\left(\left(3/2\right)-M\right)}h^{M-3/2}. \end{array}$$

The number and the values of constants *c*_1_, *c*_2_, ⋯, *c*_*N*_ depend on the *h*/*R* ratio. For example, if 0 ≤ *h*/*R* ≤ 1.1, then *Z* = *c*_1_ + (3/4)*c*_2_*R*^−1/2^*h*^1/2^ + (3/6)*c*_3_*R*^−3/2^*h*^3/2^, where *c*_1_ = 1.0220000, *c*_2_ = −0.1133000, and *c*_3_ = −0.0742000 [15].

**Figure 1:**
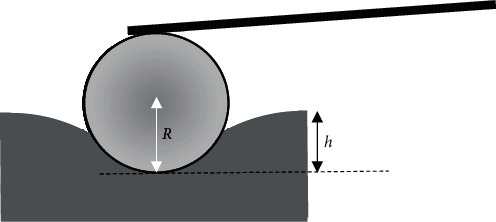
Illustration of a spherical indentation. In an indentation experiment using the presented cantilever, it is impossible to achieve a maximum indentation depth *h* > 2*R*.

As it has been previously reported, the applied force on a half space when using an axisymmetric indenter is directly proportional to the contact radius *r*_*c*_ between the indenter and the sample for a specific indentation depth [16]. In other words, *F* ~ *r*_*c*_*h*. In case of a spherical indenter with radius *R* and small indentation depths, $r_{c}=\sqrt{Rh}$; thus, *F* ~ *h*^3/2^. In case of a flat ended cylindrical indenter with radius *R*_cyl._, *r*_*c*_ = *R*_cyl._ = const.; thus, *F* ~ *h*. As a result, while the indentation depth increases when using a spherical indenter, the contact radius tends to a limit value which will be equal to the indenter's radius [15, 16]. Thus, the applied force *F* = *f*(*h*) should be at first proportional to *h*^3/2^ (very small indentation depths) and will become linear (i.e., proportional to *h*) for very big indentation depths [15, 16]. Hence, the parameter *Z* is a “correction factor” in order to apply the Hertz equation for big indentation depths (i.e., *h* > *R*/10) [15, 16]. In other words, *Z* is a mathematical quantity that accounts for the change of the slope of the *F* = *f*(*h*) curve as the indentation depth increases. Equations (4) and (6) offer a new perspective in the basic question of this paper. Under a strict mathematical way of thinking, it has been already proved that it is theoretically possible to determine simultaneously the indenter's radius and the sample's Young's modulus for very big indentation depths (*h*/*R* > 5) [15]. In this case (*h*/*R* > 5), the force indentation data becomes linear *F* = (2*ER*/(1 − *v*^2^))*h* − (*ER*^2^/(1 − *v*^2^)), and the sphere's radius can be calculated as *R* = 2*B*/*A*, where *B* is the common point of the linear fit with the *F*-axis and *A* is the slope of the linear curve [15]. Subsequently, the Young's modulus can be easily determined from the slope of the linear curve [15]. However, in real experimental procedures for big indentation depths regarding experiments on soft samples, it is possible to permanently damage the sample; thus, it is difficult to use this approach. In addition, when using AFM probes with the geometry presented in Figure 1, it is impossible to achieve an indentation depth *h* > 2*R*. Thus, the next question is if it is theoretically possible to determine *R* using small indentation depths compared to the indenter's radius (*h*/*R* < 1) and if this method is accurate for the indenter's calibration using real experimental data. In this paper, it is proved that the accurate determination of the indenter's radius even if *h* < *R* is possible, and it can be performed with accuracy when testing samples that can be approximately considered as homogeneous and isotropic. The new approach provides similar accuracy as other well-known techniques (e.g., indenter's radius measurement using an AFM calibration grating).

## 2. Materials and Methods

### 2.1. A New Method to Calculate the Indenter's Radius

Equation (4) for 0 ≤ *h*/*R* ≤ 1.1 can be expressed as follows [15]:

(7) $$\begin{array}{c} F=c_{1}\frac{4ER^{1/2}}{3\left(1-v^{2}\right)}h^{3/2}+c_{2}\frac{E}{\left(1-v^{2}\right)}h^{2}+c_{3}\frac{2ER^{-1}}{3\left(1-v^{2}\right)}h^{3}, \end{array}$$

or

(8) $$\begin{array}{c} \frac{F}{E^{*}R^{2}}=c_{1}\frac{4}{3}{\left(\frac{h}{R}\right)}^{3/2}+c_{2}{\left(\frac{h}{R}\right)}^{2}+c_{3}\frac{2}{3}{\left(\frac{h}{R}\right)}^{3}. \end{array}$$

In equation (8), *E*^∗^ = *E*/(1 − *v*^2^) is the sample's reduced modulus. Subsequently, the (*F*/*E*^∗^*R*^2^) = *f*(*h*/*R*) data was fitted to a function of the form:

(9) $$\begin{array}{c} \frac{F}{E^{*}R^{2}}=a{\left(\frac{h}{R}\right)}^{m}. \end{array}$$

It was found that the data provided by equation (8) can be accurately fitted to equation (9) (i.e., the *R*-squared coefficient results equal to 1, *R*_*s*.*c*_^2^ = 1.0000) only at the domain 0 ≤ *h*/*R* ≤ 1.1. In addition, the work done by the indenter (*W*) can be easily calculated as follows, using equation (8):

(10) $$\begin{array}{c} \frac{W}{E^{*}R^{2}}=\int_{0}^{h_{\max}}\frac{F}{E^{*}R^{2}}dh=\int_{0}^{h_{\max}}\left(c_{1}\frac{4}{3R^{3/2}}h^{3/2}+\frac{c_{2}}{R^{2}}h^{2}+c_{3}\frac{2}{3R^{3}}h^{3}\right)dh=c_{1}\frac{8}{15R^{3/2}}h_{\max}^{5/2}+c_{2}\frac{1}{3R^{2}}h_{\max}^{3}+c_{3}\frac{1}{6R^{3}}h_{\max}^{4} \end{array}$$

Thus,

(11) $$\begin{array}{c} \frac{W}{E^{*}R^{3}}=c_{1}\frac{8}{15}{\left(\frac{h_{\max}}{R}\right)}^{5/2}+c_{2}\frac{1}{3}{\left(\frac{h_{\max}}{R}\right)}^{3}+c_{3}\frac{1}{6}{\left(\frac{h_{\max}}{R}\right)}^{4}. \end{array}$$

The same procedure regarding the calculation of the work done by the indenter can be performed using equation (9), which is valid at the domain 0 ≤ *h*/*R* ≤ 1.1:

(12) $$\begin{array}{c} \frac{W}{E^{*}R^{2}}=\int_{0}^{h_{\max}}\frac{F}{E^{*}R^{2}}dh=\int_{0}^{h_{\max}}a{\left(\frac{h}{R}\right)}^{m}dh=\frac{a}{\left(m+1\right)R^{m}}h_{\max}^{m+1}=\frac{F_{\max}h_{\max}}{\left(m+1\right)E^{*}R^{2}}\Rightarrow , \end{array}$$

(13) $$\begin{array}{c} W=\frac{F_{\max}}{\left(m+1\right)}h_{\max}\Rightarrow \frac{W}{E^{*}R^{3}}=\frac{F_{\max}}{\left(m+1\right)}\frac{h_{\max}}{E^{*}R^{3}}. \end{array}$$

The work done by the indenter can be found using equation (11) or equation (13) (the results will be identical). Thus, by combining equations (11) and (13), it can be concluded as follows:

(14) $$\begin{array}{c} c_{1}\frac{8}{15}{\left(\frac{h_{\max}}{R}\right)}^{5/2}+c_{2}\frac{1}{3}{\left(\frac{h_{\max}}{R}\right)}^{3}+c_{3}\frac{1}{6}{\left(\frac{h_{\max}}{R}\right)}^{4}=\frac{F_{\max}}{\left(m+1\right)}\frac{h_{\max}}{E^{*}R^{3}}, \end{array}$$

(15) $$\begin{array}{c} m=\frac{F_{\max}h_{\max}}{E^{*}R^{3}\left[c_{1}8/15{\left(h_{\max}/R\right)}^{5/2}+c_{2}1/3{\left(h_{\max}/R\right)}^{3}+c_{3}1/6{\left(h_{\max}/R\right)}^{4}\right]}-1\Rightarrow , \end{array}$$

(16) $$\begin{array}{c} m=\frac{F_{\max}h_{\max}}{W}-1. \end{array}$$

Equation (16) can be also written in the form:

(17) $$\begin{array}{c} m=\frac{\left(F_{\max}/E^{*}R^{2}\right)\left(h_{\max}/R\right)}{W/\left(E^{*}R^{3}\right)}-1\Rightarrow , \end{array}$$

(18) $$\begin{array}{c} m=\frac{\left[c_{1}4/3{\left(h_{\max}/R\right)}^{3/2}+c_{2}{\left(h_{\max}/R\right)}^{2}+c_{3}2/3{\left(h_{\max}/R\right)}^{3}\right]\left(h_{\max}/R\right)}{c_{1}8/15{\left(h_{\max}/R\right)}^{5/2}+c_{2}1/3{\left(h_{\max}/R\right)}^{3}+c_{3}1/6{\left(h_{\max}/R\right)}^{4}}-1. \end{array}$$

**Figure 2:**
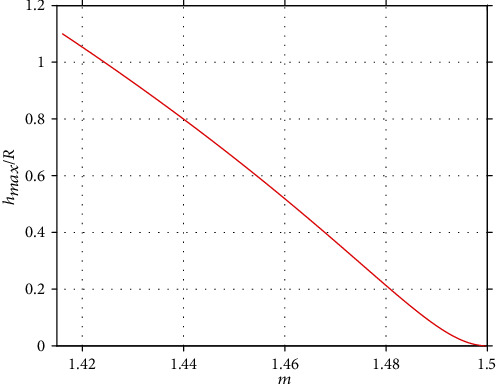
The *h*_max_/*R* = *f*(*m*) data. It can be clearly shown that for *h*_max_/*R*⟶0, *m*≅1.5. While the ratio *h*_max_/*R* increases, the exponent *m* decreases.

Although the aforementioned equation cannot be inverted, by tabulating (*m*, *h*_max_/*R*), with *h*_max_/*R* ∈ [0, 1.1] and plotting such list, the graph of the inverted function *h*_max_/*R* = *f*(*m*) can be constructed. In other words, the *m* factor is calculated using equation (18) at the domain 0 ≤ *h*_max_/*R* ≤ 1.1, and a table with two columns is constructed. The first column consists of the *m*−values, and the second column of the *h*_max_/*R*−values. Subsequently, the *h*_max_/*R* = *f*(*m*) graph is presented (Figure 2).

Thus, the value of the factor *m* can be used to reveal the *h*_max_/*R* ratio, and as a result, the value of the indenter's radius since the maximum indentation depth is a known parameter. Thus, the determination of the indenter's radius depends on the determination of factor *m* (which can be easily performed using equation (16)). In equation (16), the work done by the indenter *W* can be easily calculated using the area under the *F* = *f*(*h*) graph. In other words, under a mathematical point of view, the indenter's radius can be accurately determined using typical force indentation data by solving a simple system of two equations (equations (16) and (18)) with two unknown parameters to be determined (*m* and *h*_max_/*R*). The applied force, the maximum indentation depth, and the work done by the indenter can be easily measured using the experimental data. Subsequently, *m* can be calculated using the simple equation (16). Then, from equation (18), it is easy to calculate the only unknown parameter which is the ratio *h*_max_/*R* (the parameters *c*_1_, *c*_2_, and *c*_3_ are well defined as explained in [15]) and as a result to determine *R*. Thus, in case that the force–indentation curve is obtained on a purely linear elastic sample, the calculation of *R* is a simple mathematical problem which can be performed using equations (16) and (18) and requires only one force–indentation curve. Nevertheless, in case that the sample of interest can be approximately considered as homogeneous and isotropic over a specific range of indentation depths, the method can be also applied by using many force–indentation curves and by finding the average value of the indenter's radius as it will be discussed in the following sections.

### 2.2. Open AFM Data

Several simulated curves were used for the first testing of the new method. The AtomicJ repository [17] was used for obtaining force indentation data and calculate the spherical indenter's radius.

### 2.3. Experiments

#### 2.3.1. Sensitivity Calibration and Spring's Constant Calibration

The calibration of the probe parameters is required for accurate quantitative measurements. The applied force on the sample can be provided through Hook's law in relation to the cantilever's deflection, *F* = *kaV* (where *k* is the cantilever's spring constant, *a* is the deflection sensitivity (that converts cantilever's deflection from volt to nanometers), and *V* is the measured cantilever's deflection (in volts)) [18]. The deflection *V* is measured directly by the system's position-sensitive split photodiode detector [18]. To perform a sensitivity calibration (nm cantilever deflection per volt signal of the laser detection system), it is important to acquire a force vs. distance curve on a clean, hard surface (e.g., mica or glass) [18]. Subsequently, the deflection sensitivity *a* is determined by this force vs. distance curve by simply positioning two cursors on its contact part [18]. The spring's constant calibration was performed using the thermal noise method [18]. Of course, apart from the previously mentioned techniques, other novel and accurate methods, like the “Standardized Nanomechanical Atomic Force Microscopy Procedure (SNAP)” can be applied [19].

#### 2.3.2. Contact Point Determination

When testing soft biological samples, a significant procedure is the accurate identification of the contact point between the tip and the sample. In order to provide accurate results, the AtomicJ software was used for the identification of contact point; every point of the curve is assumed as a trial contact point, a polynomial is fitted to the precontact part, and the appropriate contact model is fitted to the force indentation data [17]. The tested point that resulted in the lowest total sum of squares is accepted to be as the contact point.

#### 2.3.3. Measurements

The measurements were performed using colloidal AFM probes (CP-PNPL-BSG-A, sQube, obtained by NanoAndMore GMBH) with spheres of nominal radius equal to 1 *μ*m. The indenters were firstly calibrated using the AFM test grating TGT1 (NT-MDT Instruments). AFM image processing was performed using the WSxM software. The experiments were conducted using agarose gels with a 2.5% concentration in a 35 mm petri dish. Agarose gels were selected since they can be approximately considered as homogeneous and isotropic. The Poisson's ratio of an agarose gel can be assumed to be equal to *v* = 0.5 due to the high-water content. Young's modulus maps on agarose gels were obtained using the AtomicJ software [17].

## 3. Results and Discussion

### 3.1. Application of the New Method on Simulated Curves

**Figure 3:**
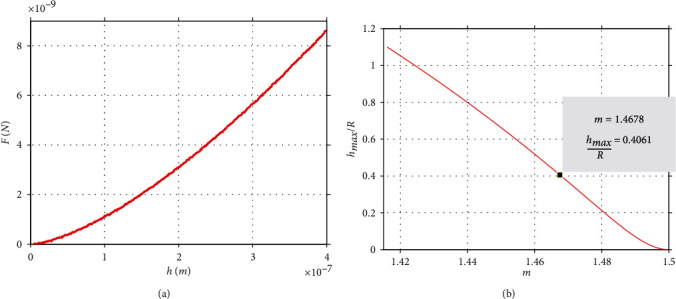
Testing on a simulated curve. (a) A simulated *F* = *f*(*h*) curve (AtomicJ repository [17]). The *h*_max_/*R* = *f*(*m*) data. It can be clearly shown that for = 1.4678⇒*h*_max_/*R* = 0.4061.

To test the validity of the new method, simulated curves (obtained from the AtomicJ repository [17] as previously mentioned in Materials and Methods) were firstly used. According to the AtomicJ repository, the simulated curves were generated in Mathematica 8.0 as if they were real force curves, using a spherical indenter. They were generated using Sneddon's relation between force and indentation depth (equation (2)), and adding random, Gaussian distributed noise. The tested sample was an elastic half-space with *E* = 20 kPa, *v* = 0.5 and the indenter's radius was  *R* = 1 *μ*m. The cantilever's spring's constant was *k* = 0.1*N*/*m*. A typical simulated curve is presented in Figure 3(a). The maximum indentation depth is 400 nm, and the maximum applied force is 8.61 nN. The area under the graph equals to *W* = 1.3967∙10^−15^ J; thus, equation (16) results in *m* = 1.4678. Using the *h*_max_/*R* = *f*(*m*) data (Figure 3(b)), it can be easily concluded that:

(19) $$\begin{array}{c} \frac{h_{\max}}{R}=0.4061\Rightarrow R=0.985\;\mu \text{m}. \end{array}$$

At this point, it must be noted that the Gaussian noise is the reason that the indenter's radius resulted slightly smaller than 1 *μ*m (this result can be easily justified since when using an indenter with radius *R* = 1 *μ*m in the settings of the AtomicJ software; the result is *E* = 20.16 kPa which is slightly bigger than 20 kPa).

### 3.2. Application of the New Method on Real Experiments

**Figure 4:**
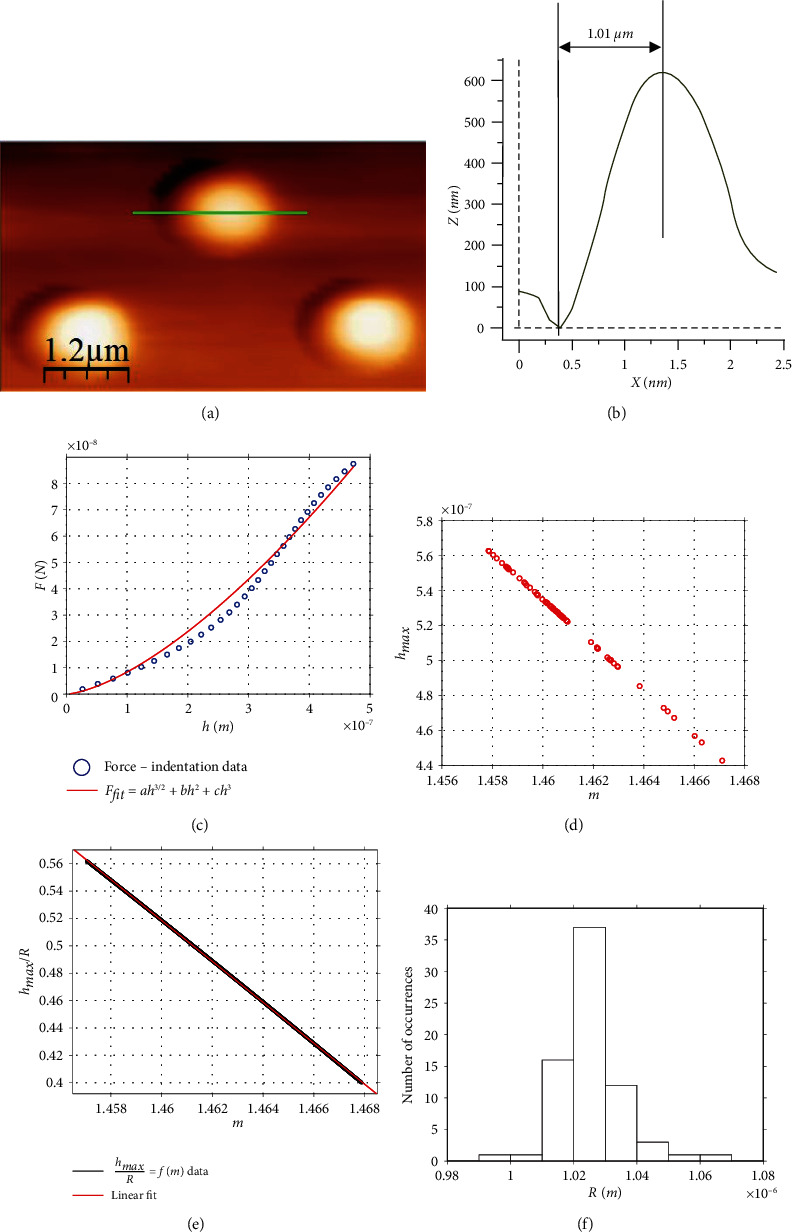
Spherical indenter's calibration. (a) Spherical indenter's calibration using an AFM grating. For (a), the PicoPlus (Molecular Imaging-Agilent, also known as 5500 Keysight Technologies system) was used in contact mode. The representative image (a) is from the topography channel so as to assess the height profile (b). (b) The sphere's radius was measured 1.01 *μ*m. (c) Force indentation data obtained on an agarose gel and a fitted curve (equation (23)). (d) The *h*_max_ versus *m* data for the 72 measurements on the agarose gel. (e) The data presented in Figure 2 at the domain 1.457 < *m* < 1.468. At the aforementioned domain, the data can be fitted to a linear equation: *h*_max_/*R* = −14.95*m* + 22.35. (f) Using the data presented in (d, e), the indenter's radius was calculated. The range of *R* values is small.

To test the validity of the proposed method in real experimental applications, hundreds of measurements on different agarose gels were obtained. The agarose gel was selected since it is approximately a soft homogeneous and isotropic material. The dimensions of a typical AFM spherical indenter that was used to conduct the experiments is presented in Figure 4(a). In Figure 4(b), the radius of the indenter is measured, *R*_meas._≅1.01 *μ*m. At this point, it is significant to note that the method presented in this paper can be applied in cases that equation (4) can be accurately used to describe the force indentation data. However, in some cases, the force indentation data may not perfectly follow equation (4). A typical example is shown in Figure 4(c). In this case, it was found that the reliability of the method is better if the real data are firstly fitted to a function of the form:

(20) $$\begin{array}{c} F_{\text{fit}}=ah^{3/2}+bh^{2}+ch^{3}. \end{array}$$

Equation (20) has the same form as equation (8) since:

(21) $$\begin{array}{c} F=c_{1}\frac{4ER^{1/2}}{3\left(1-v^{2}\right)}h^{3/2}+c_{2}\frac{E}{\left(1-v^{2}\right)}h^{2}+c_{3}\frac{2}{3}\frac{ER^{-1}}{\left(1-v^{2}\right)}h^{3} \end{array}$$

It is also significant to note that since *c*_1_ > 0 and *c*_2_ < 0, *c*_3_ < 0 it should be *a* > 0, *b* < 0, *c* < 0. Subsequently, the maximum applied force can be calculated using equation (20), i.e.,

(22) $$\begin{array}{c} F_{\text{fit}\left(\max\right)}=ah_{\max}^{3/2}+bh_{\max}^{2}+ch_{\max}^{3}. \end{array}$$

The next step is to calculate the work done by the indenter (i.e., the area under the *F*_fit_ − *h* data) and finally the factor *m* using equation (16). For example, for the case of Figure 4(c),

(23) $$\begin{array}{c} F_{\text{fit}}=280.7093h^{3/2}-23340h^{2}-1.0190∙10^{10}h^{3},R_{s.c.}^{2}=0.9820. \end{array}$$

Using equation (23), *F*_fit(max)_ = 8.499∙10^−8^*N*, *W* = 1.6318∙10^−14^ J, and *m* = 1.4630. Thus, *R* = 0.989 *μ*m. In Figure 4(d), the factor *m* with respect to the maximum indentation depth in each case (representative data from 72 measurements which were performed using the indenter shown in Figure 4(a)) is presented. The maximum indentation depth for the experiments that were performed was in the range 440 nm < *h*_max_ < 562 nm, and the range of values of *m* resulted in 1.457 < *m* < 1.468. Thus, since the *m*− values have been determined the next step was to use the *h*_max_/*R* = *f*(*m*) data at the domain 1.457 < *m* < 1.468 (Figure 4(e)).

The data presented in Figure 4(e) was fitted to a linear equation as follows:

(24) $$\begin{array}{c} \frac{h_{\max}}{R}=-14.95m+22.35,\;1.457<m<1.468. \end{array}$$

The fit was perfect since the *R*-squared coefficient resulted equal to *R*_*s*.*c*._^2^ = 1.0000. Finally, using equation (24) and the data shown in Figure 4(d), it is easy to calculate the indenter's radius:

(25) $$\begin{array}{c} R_{\text{calc}.}=1.0257\pm 0.0111\;\mu \text{m}. \end{array}$$

**Figure 5:**
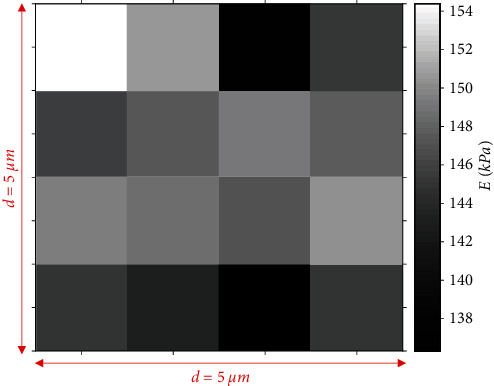
A Young's modulus map consisting of 16 measurements on an agarose gel. The Young's modulus map was created using some of the curves that were used to calculate the indenter's radius in Figure 4. Thus, it is possible to calculate the indenter's radius and the Young's modulus of the sample of interest using only a set of force indentation data. In particular, the indenter's radius can be first calculated and subsequently used to create a Young's modulus map.

The histogram displaying the range of the *R* calculations is presented in Figure 4(f). The data range is small; this fact shows the reliability of this method. In addition, it is obvious that the result was almost identical to the result obtained using the AFM calibration grating (i.e., *R*_meas._). In addition, using the mean value as calculated by the presented by this paper method (1.0257 *μ*m), a Young's modulus map which consists of 16 measurements on the same agarose gel is presented (Figure 5). Thus, it is significant to pinpoint that it is possible to determine both the spherical indenter's radius and the Young's modulus of the sample by the same measurement dataset.

**Figure 6:**
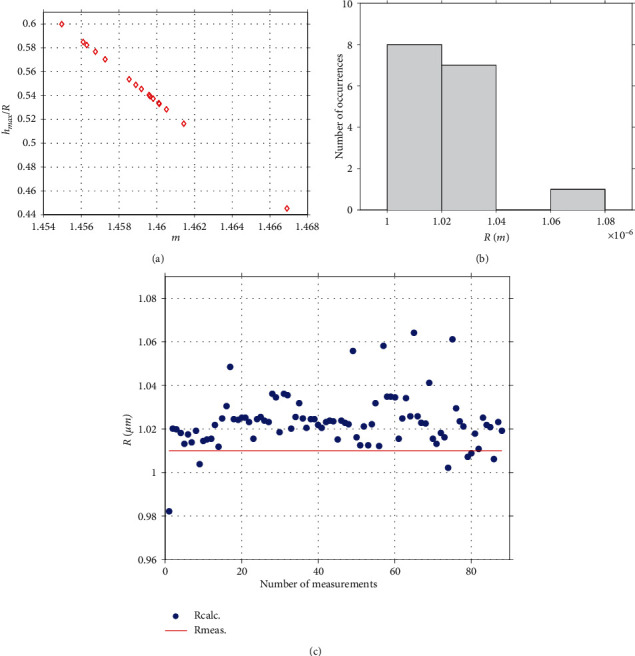
(a) The *h*_max_/*R* versus *m* data for the 16 measurements on a different agarose gel compared to Figure 4, using the same spherical indenter. (b) A histogram showing the *R* values. The mean value resulted almost identical to the case presented in Figure 4. (c) The 88 calculated values of *R* presented in histograms Figure 4(f) and (b) (blue points). The measured value using the calibration grating is also presented for comparison (red line).

In Figure 6, another one set of 16 measurements is presented which was obtained on a different agarose gel (created using the same protocol as the one used for creating Figure 4) using the same spherical indenter. In this case, the maximum indentation depth was in the range 445 nm < *h*_max_ < 600 nm. Thus, using the *h*_max_/*R* = *f*(*m*) data at the domain 1.457 < *m* < 1.468 (Figure 6(a)), it can be concluded:

(26) $$\begin{array}{c} R_{\text{calc}.}=1.0197\pm 0.0135\;\mu \text{m}. \end{array}$$

The histogram which shows the *R* values calculated from the 16 measurements is also shown in Figure 6(b). The mean ± standard deviation value is almost identical to the case of the 72 measurements. This is also a significant result since it proves that with a small number of measurements the determination of the indenter's radius can be accurately performed.

Lastly, in Figure 6(c), the 88 calculated values of *R* presented in histograms of Figures 4(f) and 6(b) and the measured value using the calibration grating (Figures 4(a) and 4(b)) are presented.

### 3.3. Reliability of the Method

**Figure 7:**
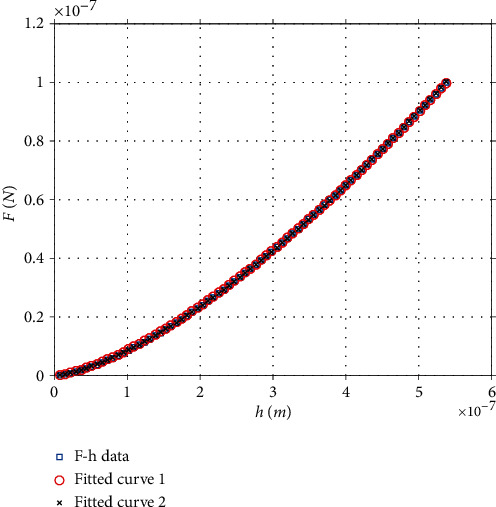
Force indentation data on an agarose gel. The data can be accurately fitted to equation (27) or equation (28). Thus, fitting the data to equation *F* = *ch*^*m*^ and determining this way, the factor *m* could result in significant errors with respect to the indenter's radius calculation. On the contrary, equation (16) provides accurate results.

From a mathematical point of view, the proposed method is rigorous and accurate, and it can be applied in every case that a homogeneous and isotropic soft sample is being tested using a perfect spherical indenter. However, many questions may arise; the first one is if it is accurate to fit the force indentation data to an equation of the form *F* = *ch*^*m*^ (where *c* = *aE*^∗^*R*^2−*m*^ according to equation (9)) and derive the factor *m* using this approach. In theory, this option seems rational; nevertheless, a significant problem arises by this approach as it is shown in Figure 7. In particular, the same force indentation data was first fitted to equation:

(27) $$\begin{array}{c} F_{\text{fit}\left(1\right)}=122.9∙h^{1.45}\;\left(S.I\right),R_{sc.}^{2}=1.0000. \end{array}$$

In this case, using equation (24), *R*_calc._ = 0.8 *μ*m.

Subsequently, the same data was fitted to equation:

(28) $$\begin{array}{c} F_{\text{fit}\left(2\right)}=153.2∙h^{1.465}\;\left(S.I\right),R_{sc.}^{2}=1.0000. \end{array}$$

In this case, *R*_calc._ = 1.2 *μ*m.

Hence, it is concluded that there are infinite combinations of the factors *c* and *m* in equation *F* = *ch*^*m*^ that can be used to perfectly fit the data. In other words, equations (27) and (28) are identical at the domain 0 ≤ *h* ≤ 538 nm. On the contrary, equation (3.2) has the major advantage that it does not depend on the parameter *c*; thus, it results in one solution in every case. For the same reasons, it is impossible to use equations (21) and (22) for the indenter's calibration. For example, a rational thought should be to fit the data to equation (20) (see the example presented in Section 3.2). Using this approach, *b* = *c*_2_(*E*/(1 − *v*^2^)) and *a* = *c*_1_(4*ER*^1/2^/3(1 − *v*^2^)). Thus, it seems that *E* and *R* can be easily calculated by solving the abovementioned set of equations. However, this is not possible since there are infinite combinations of *a*, *b*, and *c* values that could result in the exact same curve at a specific domain.

Another significant point to discuss is the selection of the maximum indentation depth. For example, for low *h*_max_/*R* values (e.g., *h*_max_/*R* = 0.1), the factor *m* will result very close to 1.5; thus, the method may not be accurately applied.

On the other hand, for very big indentation depths (e.g., *h*_max_/*R* > 1.1), the force indentation data cannot be accurately represented by a function of the form *F* = *ch*^*m*^; since as it has been previously reported when increasing the *h*/*R* ratio, the area at contact depth increases but not proportionally [15]. If *h*/*R* > 5 then, the area at contact depth is approximately equal to *A*_*c*_ = *πR*^2^, and the *F* = *f*(*h*) data become linear. In other words, the slope of the *F* = *f*(*h*) curve decreases as the *h*/*R* ratio increases [15]. It was found that only at the domain 0 ≤ *h*/*R* ≤ 1.1 the whole data range can be represented by the function (9) with accuracy. However, this is not a significant problem in most of the cases since there is no reason of conducting an indentation experiment using such big maximum indentation depths.

Following an extensive search in the literature, it was found that in practical applications, even when the indentation depth is considered to be relatively “big,” usually the case is *h*_max_ < *R*. A typical example is presented by Guo et al., who performed nanoindentation experiments on cancerous and noncancerous human mammary epithelial cells [20]. They used a spherical indenter with radius 2.65 *μ*m, and the maximum indentation depth in their experiments was 1.5 *μ*m (*h*_max_/*R* = 0.57). Another example is the Shimizu et al. publication who performed nanoindentation experiments to measure the Young's modulus of mesenchymal stem cells and HEK293 cells in the floating state [21]. In this case, *R* = 2 *μ*m and the maximum indentation depth resulted in the range 1-2.5 *μ*m. Furthermore, Sajeesh et al. calculated the Young's modulus of fibroblasts [22]. The spherical probe that was used had a radius equal to *R* = 5 *μ*m, and the maximum indentation depth in the experiments was only *h*_max_ = 1 *μ*m. The abovementioned examples indicate that there is no technical need for performing indentation experiments using very big *h*_max_/*R* ratios; even when nonhomogeneous samples are tested *h*_max_/*R* < 1 in most of the cases (other examples can be also found in [23–25]). In addition, a question that will probably arise is why a new method for the indenter's calibration is needed since the cost for acquiring a tip calibration grating is not very high. Assume that two different regions on the same agarose gel (e.g., region 1 and region 2) should be tested. Each individual experiment will probably alter the initial shape of the indenter (this could also happen during the indenter's calibration using the grating). Thus, when testing region 2, the indenter's radius will probably be different compared to the experiment on region 1. Thus, the tip radius should be retested prior experiment 2 which is of course very time consuming. In addition, the second measurement using calibration grating will probably alter (or contaminate) the indenter. However, using the proposed by this paper method, it is possible to recalculate *R* using only the force curves obtained on region 2 without remeasuring the tip radius using conventional techniques. This is very important since when testing biological samples at the nanoscale, usually 2 or 3 different regions within the same sample are being tested, with the same indenter. It is extremely time consuming to remeasure the tip radius every time.

**Figure 8:**
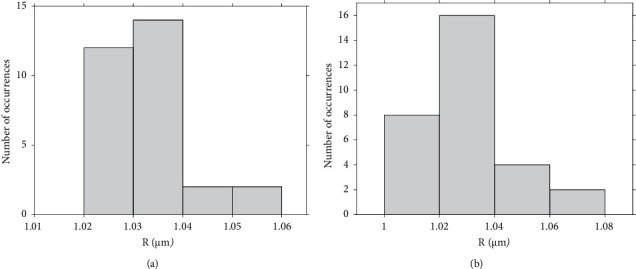
*R* measurements on two different agarose gels: (a) 30 measurements of *R* on agarose gel 1 and (b) 30 measurements of *R* on agarose gel 2.

A final test that was performed to test the reliability of this method is provided as follows. Firstly, 30 randomly selected force indentation curves were obtained on one agarose gel, and then, 30 randomly selected curves were obtained on a different agarose gel prepared with the same protocol and using the same indenter. The *R*-values which were calculated are presented in Figure 8. The null hypothesis that the two data samples are from populations with equal means was tested using a ttest2 in MATLAB. The returned value of *h* = 0 indicated that ttest2 did not reject the null hypothesis at the default 5% significance level.

It must be also noted that the proposed method can be applied to any sample regardless its stiffness under the condition that it presents approximately a linear elastic response. Despite the fact that biological samples are highly inhomogeneous, in many cases, they present a linear elastic response for a specific data range (e.g., cells) [6, 23, 26, 27]. Thus, in order to apply the proposed method for the simultaneous calculation of the sample's Young's modulus and the indenter's radius, the range of indentation values for which the sample approximately can be considered as homogeneous should be firstly determined. Subsequently, it is easy to apply the proposed method within the aforementioned data range.

### 3.4. The Effects of Errors in Spherical Indenter Calibration in Young's Modulus Determination

Since the AFM indentation method is usually used for the determination of the distribution of the Young's modulus of a soft sample, a significant point to also discuss is the effect of the possible errors in *R* calculations. For example, the presented method with respect to the 72 measurements showed in Figure 4(f) showed a 1.5% percentage difference compared to the measurement using the AFM calibration grating. Despite the fact that the measurements using the AFM grating are also not 100% accurate, assume that the real value of the indenter's radius is 1.01 *μ*m and the presented by this paper calculations resulted in 1.0257 *μ*m. The error in Young's modulus calculation in this case can be calculated using equation (8) in the following form:

(29) $$\begin{array}{c} E=\frac{F\left(1-v^{2}\right)}{R^{2}\left[c_{1}4/3{\left(h/R\right)}^{3/2}+c_{2}{\left(h/R\right)}^{2}+c_{3}2/3{\left(h/R\right)}^{3}\right]}. \end{array}$$

In particular, assuming that *E*_1_ is the Young's modulus using *R*_meas._ = 1.01 *μ*m and *E*_2_ is the Young's modulus calculation using *R*_calc._ = 1.0257 *μ*m, the ratio |*E*_1_ − *E*_2_|/*E*_1_ is presented as follows:

(30) $$\begin{array}{c} \frac{\left|E_{1}-E_{2}\right|}{E_{1}}100\%=\frac{\left(1/R_{\text{meas}.}^{2}\left[c_{1}4/3{\left(h/R_{\text{meas}.}\right)}^{3/2}+c_{2}{\left(h/R_{\text{meas}.}\right)}^{2}+c_{3}2/3{\left(h/R_{\text{meas}.}\right)}^{3}\right]\right)-\left(1/R_{\text{calc}.}^{2}\left[c_{1}4/3{\left(h/R_{\text{calc}.}\right)}^{3/2}+c_{2}{\left(h/R_{\text{calc}.}\right)}^{2}+c_{3}2/3{\left(h/R_{\text{calc}.}\right)}^{3}\right]\right)}{1/R_{\text{meas}.}^{2}\left[c_{1}4/3{\left(h/R_{\text{meas}.}\right)}^{3/2}+c_{2}{\left(h/R_{\text{meas}.}\right)}^{2}+c_{3}2/3{\left(h/R_{\text{meas}.}\right)}^{3}\right]}100\%. \end{array}$$

**Figure 9:**
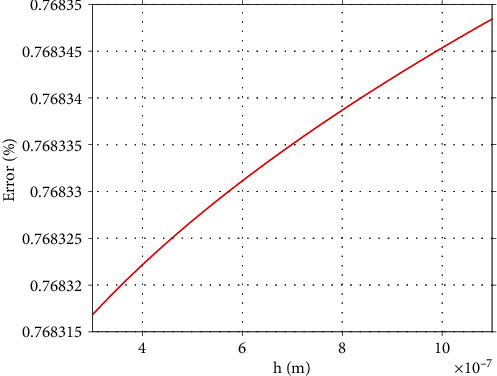
The order of magnitude of possible errors. The percentage error in Young's modulus calculation assuming that the value of the indenter that is used for calculations is 1.0267 *μ*m and the real value of the indenter's radius equals to 1.01 *μ*m (as calculated using the AFM calibration grating).

In Figure 9, the percentage error as calculated using equation (30) is presented. The domain that was used was 300 nm ≤ *h* ≤ 1100 nm. The error was calculated at the domain:

(31) $$\begin{array}{c} 0.768315\%\leq \frac{\left|E_{1}-E_{2}\right|}{E_{1}}100\%\leq 0.768348\%. \end{array}$$

For small indentation depths (*h*_max_ ≪ *R*),

(32) $$\begin{array}{c} \frac{\left|E_{1}-E_{2}\right|}{E_{1}}100\%=\frac{\left(1/R_{\text{meas}.}^{1/2}\right)-\left(1/R_{\text{calc}.}^{1/2}\right)}{1/R_{\text{meas}.}^{1/2}}100\%=0.7683\%, \end{array}$$

according to equation (1). Thus, for big *h*_max_/*R* ratios, the percentage error is slightly bigger. An error at the range 0.7%-0.8% is negligible since it is smaller compared to the error provided in Young's modulus calculation if the Hertz equation (1) is used for an indentation experiment using a spherical indenter for *h*_max_/*R* = 0.1 (which is the generally accepted limit for using equation (1) [11]). In this case (i.e., *h*_max_/*R* = 0.1), the factor *Z* in equation (6) results in [13]:

(33) $$\begin{array}{c} Z=0.9905. \end{array}$$

Thus, the percentage error in this case is:

(34) $$\begin{array}{c} \frac{\left|E_{\text{hertz}}-E_{\text{accurate}}\right|}{E_{\text{accurate}}}100\%=\frac{\left|1-\left(1/Z\right)\right|}{1/Z}100\%\approx 1\%. \end{array}$$

### 3.5. Summarizing the Steps of the Method

In this paper, a new method for the calibration of spherical indenters, directly from force–indentation curves, was presented and discussed. The major advantage of this method is that it can be used to simultaneously calculate the spherical indenter's radius and the sample's Young's modulus (in the case that the sample can be considered as homogeneous and isotropic) using a typical set of force–indentation curves. In addition, it can be used even if the data do not perfectly follow equation (4). In this case, the data can be fitted to equation (20), and the method can be equally applied using the fitted curve without reducing the accuracy. The steps that should be followed for the determination of the indenter's radius with accuracy are summarized as follows:

- An approximately homogeneous and isotropic material (with unknown Young's modulus) such as an agarose gel should be used as a reference sample. Since the range of *R* was relatively small as presented in Figure 4(f), there is no need for processing hundreds of force indentation curves. Probably, 15-20 curves are enough to conclude in an accurate calculation. However, the range of *R* should be tested to conclude if the result is accurate; the standard deviation should be small. A standard deviation equal to the 10% of the average value should be acceptable (i.e., *R*_calc._ = *R*_ave_ ± (10/100)*R*_ave_). In this case, the error regarding *R* calculation should be approximately up to 10%. Assume for example a 10% error in the indenter's radius compared to the real value (i.e., *R*_calc._ = 1.1 *μ*m, assuming that the real value is 1 *μ*m). In this case, the percentage error in the Young's modulus calculation according to equation (30) will be approximately 4.65% (for 0 ≤ *h* ≤ 1.1 *μ*m) which is comparable to other systematic errors regarding AFM experiments [28]
- The data should be fitted to the following equation (in case that the data do not accurately follow equation (4)):

(35) $$\begin{array}{c} F_{\text{fit}}=ah^{3/2}+bh^{2}+ch^{3},a>0,b<0,c<0. \end{array}$$

(iii) Using the area under the fitted curve (which equals to the work done by the indenter), the maximum force (i.e., *F*_fit(max)_) and the maximum indentation depth in the experiment, the factor *m* can be calculated:

(36) $$\begin{array}{c} m=\frac{F_{\max}h_{\max}}{W}-1. \end{array}$$

In case that *m* < 1.41 or *m* is close to 1.5, the measurement should be repeated since for very big indentation depths the method is not valid, and for small indentation depths, the accuracy is low

(iv) Using the data presented in Figure 2 (resulted using equation (18)), for each value of the factor *m*, the *h*_max_/*R* ratio can be easily calculated. Thus, since *h*_max_ is a known parameter in each case, *R* can be calculated
(v) The mean ± standard deviation of *R* is calculated, and the histogram of *R* values should be also constructed to evaluate the range of *R* values. The mean *R* equals to the indenter's radius. The histogram should be also used to conclude whether the range of values is small or not. In case of extensive range of *R* values, the calibration should be repeated

**Figure 10:**
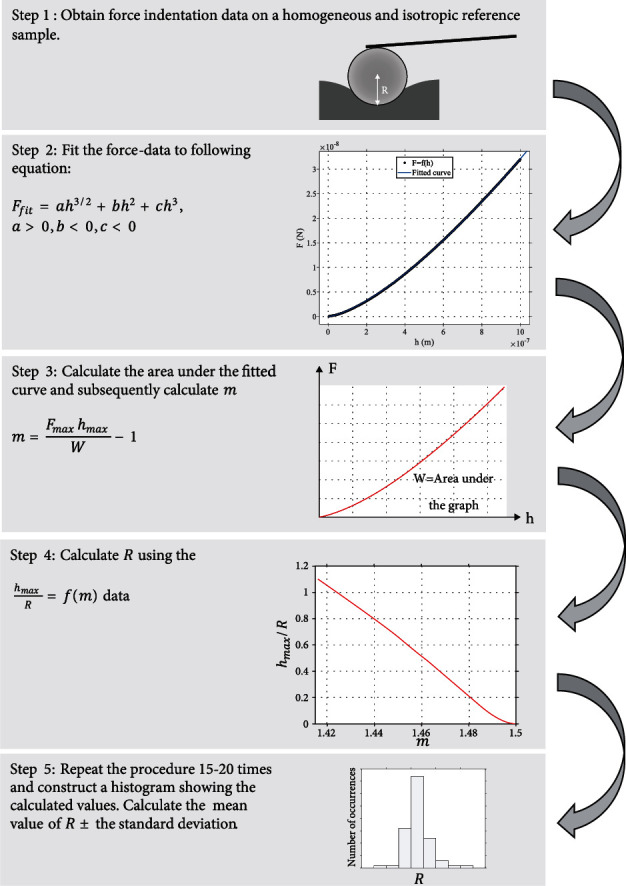
The steps for the indenter's calibration. The 5 basic steps towards the calculation of a spherical indenter's radius are presented.

The steps of the method are also summarized in Figure 10. The significance of the presented work has many different aspects. Firstly, the interesting mathematical question whether it is theoretically possible to calculate *E* and  *R* using the same force indentation data (regarding spherical indentations) was answered. In addition, it was shown that this method can be applied easily in real AFM experiments. This fact is extremely important since it reduces the required equipment to perform an AFM indentation experiment (calibration gratings or SEM imaging is no longer required). Furthermore, the ability to simultaneously calculate the indenter's radius and the Young's modulus of a homogeneous and isotropic soft material is also important since it significantly reduces the time, the experimental effort, and the budget of the experiment. AFM indentation is a powerful method for the mechanical characterization of biological samples at the nanoscale. The applications of the method are numerous especially in medical diagnosis (e.g., cancer diagnosis) [29–31]. Thus, the ability to simplify the experimental procedures may increase the possibility of applying the method in real clinical applications.

## 4. Conclusion

A method for calibrating spherical indenters used in AFM indentation experiments regarding soft biological samples was presented and discussed. Firstly, it was shown that it is mathematically possible to simultaneously calculate the indenter's radius and the Young's modulus of a sample that behaves like an elastic half space. In addition, it was also shown that the presented method can be used in real indentation experiments. The results obtained by measurements on agarose gels showed that the accuracy of the method is comparable to the accuracy provided by calibration using AFM gratings.

## Data Availability

All the data are included in the manuscript.
